# Effects of weighted vests *versus* elastic bands plyometric jump training on lower-body physical performance adaptations in volleyball players

**DOI:** 10.3389/fphys.2026.1756391

**Published:** 2026-03-04

**Authors:** Yichen Bao, Yuankai Qiao, Shuowen Yang

**Affiliations:** Department of Physical Education, Liaoning Normal University, Dalian, Liaoning, China

**Keywords:** athletic performance, ball sports, bio-motor abilities, physical conditioning, sport-specific training

## Abstract

**Introduction:**

The aim of this study was to investigate the impact of different plyometric jump training (PJT) loading strategies, using either weighted vests (WV) or elastic bands (EB), on the physical and physiological performance of young volleyball players.

**Methods:**

Thirty-two male volleyball players participated in the study and were randomly assigned to WV-PJT (n = 8), EB-PJT (n = 8), unloaded PJT (UL-PJT, n = 8), and a control group (CG, n = 8). The players in the WV and EB groups performed PJT with resistance equivalent to 10% of body mass, while the UL group engaged in PJT without any additional loads. Moreover, the players in the CG only performed regular volleyball practice. Countermovement vertical jump (CMVJ), 10-m sprint, T-test change of direction speed (T-CODS), reactive strength index (RSI), 1 repetition maximum of leg press (1RM_LP_), and Wingate anaerobic power tests were measured before and after the 8-week training period. A two-way repeated measures analysis of variance (ANOVA) (4 [group] x 2 [time]), followed by Bonferroni *post hoc* testing, was employed to identify any significant differences in pairwise comparisons.

**Results:**

All training groups (i.e., WV-PJT, EB-PJT, and UL-PJT) showed significant improvements (p = 0.001) in physical and physiological performance outcomes, with effect sizes ranging from small to large over the training period. Both the WV-PJT and EB-PJT groups exhibited greater adaptive changes (p < 0.05) compared to the UL-PJT group after the intervention. For the 1RM_LP_, the WV-PJT group demonstrated significantly greater adaptive responses (p = 0.011, standard mean difference [SMD] = 0.51, 95% CI = −0.51–1.48) than the EB-PJT group. Conversely, the EB-PJT group showed greater adaptations in the RSI (p = 0.041, SMD = 0.46, 95% CI = −0.53–1.45), as well as in peak power (p = 0.006, SMD = 0.49, 95% CI = −0.53–1.46) and mean power output (p = 0.017, SMD = 0.33, 95% CI = −0.67–1.30) compared to the WV-PJT group.

**Conclusion:**

Loaded PJT is an effective method for producing greater adaptations in volleyball players. Specifically, WV-PJT is more beneficial for strength gains, while EB-PJT is superior for improving RSI and anaerobic power output.

## 1 Introduction

The physical performance of volleyball players is significantly influenced by their jumping ability, encompassing both vertical movements (e.g., spike and serve jumps) and horizontal jumps (e.g., block jumps). While vertical jumps are crucial for attacking and blocking, horizontal jumps are essential for defensive maneuvers and court coverage (Pisa et al., 2022). Additionally, muscular strength and power are crucial for players to achieve optimal force production during spiking and serving in a match (Oliveira et al., 2025). Players in a volleyball match usually perform over 150 jumps, highlighting the importance of explosive muscular power, strength, and anaerobic power in improving scoring actions such as spiking, blocking, and serving (Silva et al., 2019; Ning and Sheykhlouvand, 2025). Therefore, it is crucial to employ appropriate training techniques that not only enhance the physical performance (i.e., strength, speed, and jumping ability) but also improve the anaerobic power output in volleyball players.

Although there are various training methods recommended for volleyball players, such as strength training (Keoliya et al., 2024; Arazi et al., 2018), sprint interval training (Tao et al., 2024; Kaynak et al., 2017), and complex training (Wang et al., 2023), recent review and experimental studies indicated that plyometric jump training (PJT) is an effective training modality to enhance jumping ability, sprint performance, anaerobic power output, and muscular strength for young male and female volleyball players with different fitness levels (Silva et al., 2019; Ramirez-Campillo et al., 2020; 2021; Hernandez-Martinez et al., 2023). PJT is a form of neuromuscular training designed to enhance explosive power by improving the ability of the muscle–tendon unit to rapidly produce force (Junior and Pinillo, 2025; Ramirez-Campillo et al., 2020; Chu, 1998). It involves a repetitive sequence of movements in which each cycle includes a rapid deceleration of the body (eccentric phase), immediately followed by a brief transition phase (amortization phase), and a rapid acceleration in the opposite direction (concentric phase) (Junior and Pinillo, 2025; Silva et al., 2019; Chu, 1998). This rapid coupling of eccentric and concentric muscle actions engages the stretch-shortening cycle (SSC), whereby elastic energy storage and neural reflex activation during the eccentric phase enhance force production during the subsequent concentric action (Chu, 1998), ultimately leading to improvements in lower-body physical performance in athletes (Ramirez-Campillo et al., 2021).

To create an appropriate PJT program, it is crucial to take into account several variables, including the number of sets, repetitions, intensity, types of exercises, rest durations, and the training surface (Saez de Villarreal et al., 2009; 2010). Nevertheless, a crucial factor that has been overlooked in previous research is the role of additional loads, such as dumbbells, barbells, or weighted vests, during the jump training. In fact, implementation of external loads has been suggested as an effective strategy in PJT to enhance anaerobic power output as well as physical performance in players (Stankovic et al., 2025; Iranpour et al., 2025; Ali et al., 2024; Khlifa et al., 2010; Kobal et al., 2017; Kang, 2018; Freitas-Junior et al., 2019; Aloui et al., 2020; Aloui et al., 2021; Gaamouri et al., 2023; Novak et al., 2023). Although PJT is traditionally performed using body weight alone (Ramirez-Campillo et al., 2020; Hernandez-Martinez et al., 2023; Saez de Villarreal et al., 2010), previous studies demonstrated that incorporating external loads, such as weighted vests (WV) (Ali et al., 2024; Khlifa et al., 2010; Kobal et al., 2017) or elastic bands (EB) (Stankovic et al., 2025; Aloui et al., 2020; Aloui et al.,2021), during the PJT can enhance neuromuscular properties and potentially greater physical performance adaptations.

The utilization of a WV during PJT can increase the eccentric load on the lower extremities, potentially leading to enhanced neuromuscular adaptations and an increased internal training load (Iranpour et al., 2025; Ali et al., 2024; Kang, 2018). Eccentric muscle actions, such as the lowering phase of a jump, generate force while lengthening, and an increased eccentric load places greater demands on muscle fibers and the surrounding connective tissues (Chu, 1998). This stimulus drives adaptations including increased muscle stiffness, improved force-conversion efficiency, and enhanced muscle damage leading to subsequent repair and hypertrophy (Ali et al., 2024). Furthermore, the incorporation of WV in PJT has been shown to elicit greater neuromuscular adaptations and enhanced physical performance compared to training without added loads (Iranpour et al., 2025; Khlifa et al., 2010). Unlike WV, EB offer an alternative method of loading during PJT by providing variable resistance across the range of motion. Specifically, EB resistance increases progressively as the elastic bands elongate during the early-to-mid concentric phase of the jump, when joint angular velocities are rising and force and power demands are near their peak. As the movement approaches full hip, knee, and ankle extension at take-off, band elongation reaches its maximum and subsequently decreases, resulting in a reduction in elastic tension and external resistance toward the end of the concentric phase (Stankovic et al., 2025). This variable resistance profile can meaningfully influence the SSC and the rate of force development, thereby promoting improvements in reactive strength and jump performance (Stankovic et al., 2025; Aloui et al., 2020). By progressively increasing mechanical demands across the range of motion, EB loading provides an enhanced stimulus to the neuromuscular system, particularly by augmenting motor unit recruitment and rate coding, which are key mechanisms underlying explosive performance adaptations (Stankovic et al., 2025; Aloui et al., 2021). Additionally, incorporating elastic resistance during training sessions not only enhances the eccentric stimulus but also increases myoelectric activity in the relevant musculature (Aloui et al., 2020). This heightened myoelectric activity contributes to enhanced adaptive responses and improved physical performance, specifically in areas such as jumping, sprinting, change of direction speed, and strength (Gaamouri et al., 2023). Therefore, we can utilize two strategies to incorporate additional loads: (a) maintaining a constant load with PJT using the WV determined by a percentage of body mass (e.g., 10% of body mass) (Kang, 2018), or (b) variable loads using EB during PJT to ensure consistent loading throughout the range of motion (Gaamouri et al., 2023).

Some suggestions have been made regarding the limited effectiveness of WV in stimulating the neuromuscular system during exercises with an ascending strength curve (Kang, 2018). During these exercises, the force produced by the involved muscle groups generally increases throughout the concentric phase of the movement, peaking near the end of the range of motion (Khlifa et al., 2010; Kobal et al., 2017). Conversely, when using EB, the load appears to be distributed more evenly throughout the entire range of motion of the exercise (Aloui et al., 2021). There is a suggestion that EB may have an advantage in facilitating muscles to produce near-maximal force throughout the range of motion (Gaamouri et al., 2023). However, further longitudinal research is necessary to gain a complete understanding of the role of WV versus EB in relation to the incorporation of added loads in PJT programs to clarify the promising suggestions.

To date, there have been no studies that have specifically examined the effects of using WV versus EB during PJT on the physical performance of volleyball players. Additionally, the superiority of each additional load strategy during PJT on physical and physiological performance adaptations for male volleyball players remains unknown. Therefore, this study aimed to investigate two main aspects: (a) the effects of PJT on the physical and physiological performance (i.e., jumping ability, sprint and change of direction speed, muscular strength, reactive strength index, and anaerobic power output) of volleyball players, and (b) the comparison between two different loading strategies—WV (constant load) and EB (variable load)—in maximizing training adaptations in male volleyball players. We hypothesized that PJT could be an effective training modality for enhancing physical performance, specifically in areas such as jumping, sprinting, change of direction speed, and strength, assuming that the inclusion of extra loads during PJT would provide additional benefits in terms of neuromuscular adaptations, ultimately leading to greater gains in physical performance for volleyball players.

## 2 Methods

### 2.1 Estimation of sample size

In order to determine the suitable sample size for this study, the study referred to the research conducted by Freitas-Junior et al. (2019) who examined the effects of loaded and unloaded PJT in male volleyball players. To calculate the sample size, G*Power software (Version 3.1.9.2, University of Kiel, Germany) was utilized, with effect size of 0.21, a power of 0.8, and a significance level of 0.05. It was established that 6 subjects per group were required for this study. However, to account for potential participant dropout during data collection, the sample size was subsequently increased to eight participants per group with the same playing position as follows: one setter, two middle blockers, three outside hitters, and two opposite hitters.

### 2.2 Subjects

Thirty-two young male volleyball players from a single volleyball academy volunteered to participate in this study. All participants had similar training habits and comparable volleyball training experience and were considered *trained* athletes (i.e., Tier 2) (McKay et al., 2021). The players were divided into three training groups: weighted vests PJT (WV-PJT), elastic bands PJT (EB-PJT), and unloaded PJT (UL-PJT), as well as a control group (CG), with each group comprising eight subjects (Table 1). Prior to their participation in the study, the players were already familiar with the PJT method and had refrained from engaging in this type of training for a minimum of 6 months. The exclusion criteria for the study included players with potential medical issues, recent ankle, knee, or back problems, medical or orthopedic conditions that could impact their involvement or performance, subjects who had undergone upper or lower extremity reconstructive surgery within the past 2 years, or those with unresolved musculoskeletal disorders (Gharaat et al., 2025). Approval for the study was granted by the Ethics Committee of Liaoning Normal University, and the athletes provided informed consent prior to the commencement of the research. The study was conducted according to the ethical guidelines outlined in the Declaration of Helsinki.

**TABLE 1:** Descriptive data (mean ± SD) of the subjects.

Characteristics	WV-PJT	EB-PJT	UL-PJT	CG
Age (y)	20.9 ± 1.4	20.7 ± 1.7	20.6 ± 1.2	20.4 ± 1.1
Height (cm)	183.4 ± 5.1	182.8 ± 4.5	183.2 ± 4.1	183.8 ± 3.6
Body mass (kg)	79.8 ± 3.2	79.4 ± 3.1	78.8 ± 3.3	79.6 ± 4.1

### 2.3 Study design and procedure

**FIGURE 1:**
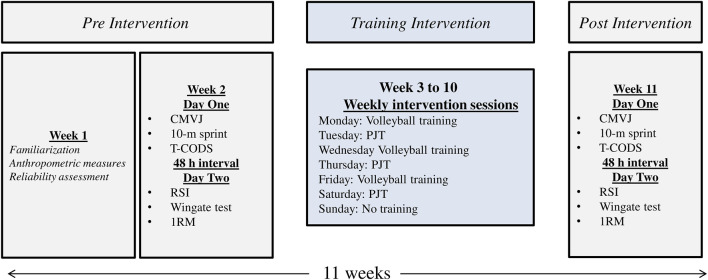
Study design.

The study investigated the impact of an 8-week PJT using varied strategies for additional loads (WV or EB) on the physical (jumping ability, sprinting speed, change of direction ability, lower-body maximal strength, and reactive strength index) and physiological performance (anaerobic power output) adaptations of male volleyball players during the off-season. The study utilized a parallel four-group design for a duration of 11 weeks from October to December. One week was allocated for familiarization and reliability assessment, followed by 1 week for pre-testing. During the pre-test, jump, sprint, and change of direction abilities were assessed on day one (Monday), while reactive strength index, Wingate anaerobic power, and strength tests were conducted on day two (Wednesday). Participants then completed 8 weeks of training. Post-testing was performed during the final week, with the same distribution and order of tests as the pre-test, to ensure consistency (Figure 1). All tests were administered by the same experienced strength and conditioning specialist to ensure consistency across testing sessions, and all testing procedures were supervised by the research team throughout all testing phases. All subjects were provided with specific instructions prior to the test day. These instructions encompassed several key aspects: 1) ensuring a minimum sleep duration of 9 hours, 2) consuming the prescribed amount of carbohydrates and maintaining proper hydration, and 3) abstaining from the use of drugs or performance-enhancing supplements. Prior to testing, all participants completed a standardized 15-min warm-up: 5 minutes of low-to moderate-intensity jogging (≈60–70% of maximal heart rate), 5 minutes of dynamic stretching (leg swings, walking lunges, high-knee marches), and 5 minutes of sport-specific drills (short accelerations, bounding, and vertical jumps). Thereafter, two to three practice trials of each test were performed as a specific warm-up. The physical performance assessments (i.e., jumping ability, sprinting speed, change of direction ability) took place on a volleyball wooden court, with temperatures ranging from 26 °C–28 °C. Furthermore, laboratory-based measurements including reactive strength index, anaerobic power output and maximal strength were conducted in a controlled environment with temperatures ranging from 25 °C–27 °C and humidity levels between 45%–50%. To eliminate any potential influence of footwear, all players were instructed to wear the same shoes during both the pre and post-tests. Additionally, all training and testing sessions were scheduled in the afternoon. Reliability was evaluated using intraclass correlation coefficients (ICC), based on a two-way random-effects model with absolute agreement, with 95% confidence intervals. Two measurements were obtained from a subgroup of 10 volleyball players (age: 20.5 ± 1.1 years; height: 182 ± 4 cm; body mass: 79.1 ± 3.1 kg) with a 48-h interval between sessions.

### 2.4 Measurements

#### 2.4.1 Anthropometry

A wall-mounted stadiometer (Seca 222, Terre Haute, IN) with a precision of 0.5 cm was employed to determine the height of the subjects. For evaluating the body mass of the players, a digital scale (Tanita BF683W scales, Munich, Germany) with an accuracy of 0.1 kg was utilized (Ning and Sheykhlouvand, 2025).

#### 2.4.2 Jump and reactive strength performance

In this study, the countermovement vertical jump (CMVJ) was used to assess jump performance in volleyball players and was evaluated using a Vertec device (Power Systems, Knoxville, TN, United States). Players were instructed to perform a rapid countermovement with a natural and unrestricted arm swing throughout both the downward and upward phases of the movement, immediately followed by a maximal-effort vertical jump. All jumps were initiated with both feet in contact with the ground, and participants used their dominant hand to displace the highest reachable Vertec vane. Upon landing, players were required to maintain an upright posture and flex their knees to ensure a safe and controlled landing. Each participant performed three maximal trials, and the highest valid jump was retained for subsequent analysis. Jump height was calculated as the difference between standing reach height and the highest vane reached during the jump (Gehri et al., 1998). The ICC with 95% CI for CMVJ was 0.95 (95% CI = 0.80–0.99). The players executed depth jumps from a 45-cm high platform, using an electronic contact mat system (Globus Tester, Codogne, Italy) with a precision of 0.01 m to measure the reactive strength index (RSI), which was previously described in detail (Ramirez-Campillo et al., 2018). They were instructed to place their hands on their hips, step off the platform with the leading leg straight, and ensure a drop height of 45 cm by avoiding any initial upward propulsion. The objective was to jump for maximal height and minimal contact time in order to maximize jump reactive strength. Three repetitions were carried out, with 15 s of rest between repetitions and the most successful trial was chosen for subsequent statistical analysis. The ICC with 95% CI for RSI was 0.97 (95% CI = 0.87–0.99).

#### 2.4.3 Sprint and change of direction speed

The players’ sprinting ability was assessed over a 10-m distance. The test involved starting from a crouched position and beginning the sprint upon hearing a “GO” sound. Infrared beams were set up at the 10-m mark, positioned 1-m above the ground (at hip level), and measured using a photoelectric cell (Globus Tester, ±0.01 s). Three trials were conducted with a 1-min rest interval between each trial, and the trial with the best performance was selected for further statistical analysis (Gjinovci et al., 2017). The ICC with 96% CI for 10-m sprint was 0.97 (95% CI = 0.86–0.98). To assess the change of direction speed (CODS) of volleyball players, the T-test (T-CODS) was used. This assessment was conducted to evaluate speed by focusing on directional changes such as forward sprinting, side shuffling to the left and right, and backpedaling (Miller et al., 2006). Players were given instructions to start from a standing sprint position and sprint towards a cone placed 10-m away. Following that, they had to perform a side-shuffle to a cone positioned 5-m away. After touching the cone, players were required to side-shuffle to a cone located 10-m to the right, and then return to the middle cone using the same side-shuffling technique. The test concluded with players backpedaling to the starting line. The best time out of three attempts was recorded as the test score, with a 3-min rest interval provided between each trial. Players would be disqualified if they failed to touch any of the cones, crossed one foot in front of the other, or did not face forward throughout the entire test. Infrared beams were located at the end point, positioned 1-m above the ground, for the purpose of collecting measurements with a photoelectric cell. The ICC with 95% CI for T-CODS was 0.94 (95% CI = 0.84–0.96).

#### 2.4.4 Lower-body maximal strength

In this study, the one repetition maximum of leg press exercise (1RM_LP_) (Body Solid, GLPH 1100, United States) was used to assess lower-body maximal strength of players. The 1RM_LP_ was conducted using the method described in detail by Kraemer and Fry (1995). Briefly, the players are positioned in a seated posture (with approximately 120° of hip flexion, 80° of knee flexion, and 10° of dorsiflexion), while the load moves diagonally at a 45° angle. Upon receiving the “GO” command, the players executed a concentric leg extension. The extension began from a flexed position of 80° and continued until reaching the full extension of 180° in the knees while facing the resistance determined by the weight. Prior to this exercise, a warm-up routine was performed, consisting of a set of five repetitions at 50%–60% of the estimated maximum weight. This weight was estimated using a combination of the player’s self-reported 1RM and their recent training load. Detailed instructions were provided to the subjects on how to properly execute the 1RM_LP_ test, emphasizing correct form and ensuring a complete range of motion. Specific form cues included maintaining a neutral spine throughout the movement, executing a controlled descent to at least mid-thigh, and achieving full hip extension at the top of the repetition. These cues were aligned with established biomechanical principles for optimal exercise performance (Kraemer and Fry, 1995). The 1RM test was conducted by gradually increasing the load in successive trials until the subjects reached their maximum weight within the appropriate range of motion. In total, three to five attempts were made to determine the 1RM_LP_, with a 3-min break allowed between each trial to facilitate adequate recovery. The ICC with 95% CI for 1RM_LP_ was 0.93 (95% CI = 0.76–0.98).

#### 2.4.5 Wingate anaerobic power

The study measured the peak power output (i.e., the highest power achieved during the 5-s mark) and mean power output (i.e., the average power throughout the test) of the lower body by conducting a 30-s maximal Wingate test on a mechanically braked cycle ergometer (model 894E, Monark, Sweden). After a 5-min warm-up on the cycle, players pedaled at their maximum speed against the resistance of the device, with an extra load equivalent to 0.075 kg kg^–1^ of their body mass. Verbal motivation was provided to ensure subjects exerted maximum effort during the 30-s test (Kasabalis et al., 2005).

#### 2.4.6 Training intervention

The players maintained their usual volleyball training regimen, which consisted of tactical exercises, technical drills, and practice matches on Monday, Wednesday, and Friday afternoons, with each session lasting approximately 90–100 min. The PJT program was conducted 3 days per week (i.e., Tuesday, Thursday, and Saturday), with a minimum of 48 h of rest between each session, for a total of 8 weeks. Each session lasted between 65 and 70 min. Prior to the main part of the training session, there was a standard warm-up lasting 10 minutes including 5 minutes of submaximal running, 5 minutes of stretching exercises, and 10 submaximal vertical jumps. The PJT consisted of squat jumps, depth jumps from a 45-cm box, and a 45-cm height box jumps (i.e., a vertical jump onto a stable box, followed by stepping down in a controlled manner; jumping down was not permitted) (Chu, 1998). A 45-cm box height was chosen based on existing literature demonstrating its effectiveness in maximizing power output and vertical jump performance, particularly for volleyball players (Liu et al., 2025; Ducrocq et al., 2020). In order to mitigate the adverse impact of landing during PJT, a five-cm mat was utilized to minimize strain on the musculoskeletal system. The number of repetitions gradually increased from week one to week eight, as shown in Table 2 (Asadi et al., 2016). The CG did not participate in any training intervention but underwent the same testing protocols as the other groups. The training took place on a wood volleyball court, and the players were instructed to jump for maximum height and minimum contact time in each jump. A researcher was present during the training sessions to motivate the players to give their maximum effort. Additionally, a specialized strength and conditioning coach closely supervised the players throughout the sessions with a coach-to-player ratio of 1:4.

**TABLE 2:** Gradual increases in the number of repetitions through an 8-week intervention.

Training week	Set	Repetition
W1	3	8
W2	3	9
W3	3	10
W4	3	11
W5	4	9
W6	4	10
W7	4	11
W8	4	12

In UL-PJT, the players underwent the training program by using their body mass. Conversely, the WV-PJT group performed the training exercises with a weight vest (LEKARO, FZ-4685) that was equal to 10% of their body masses. This specific percentage of body mass has been identified as an effective load for enhancing the rate of force development in the lower extremities during PJT (Kang, 2018). Additionally, the EB-PJT group took part in the training program utilizing an elastic band system (Thera-Bands, Akron, United States) that included four latex bands with strong elasticity in black color (250% elongation equivalent to 8 kg load) (Aloui et al., 2021). To create additional loads during the EB-PJT, a vest was used to attach an EB to the player’s body on one end, while the other end was fixed to the device. These elastic bands provided resistance throughout the range of motion, referred to as variable loads. Although there were differences between the WV and EB methods, attempts were made to equalize the loads among the groups. For the intervention, a WV equivalent to 10% of the body mass (∼8 kg) and a black EB representing approximately 8 kg loads was employed for the players to compare the effects of constant or variable loads through PJT in volleyball players.

### 2.5 Statistical analysis

The data analysis was conducted using SPSS software (Version 24, SPSS Institute in Chicago, IL, United States). The values were expressed as mean ± standard deviations (SDs). The normality of the data was assessed using the Shapiro-Wilk test for both pre-test and post-test values, while Levene’s test was employed to evaluate the homogeneity of variance. Subsequently, the data was analyzed using a two-way repeated measures ANOVA (4 [group] x 2 [time]) (Gharaat et al., 2024), and the Bonferroni *post hoc* test was used to identify any significant differences in the pairwise comparisons to control type 1 error (Arazi et al., 2018). The assumption of sphericity was evaluated using Mauchly’s test, and when violations were detected, degrees of freedom were adjusted using the Greenhouse–Geisser correction. The effect size (ES) with a 95% CI was utilized to evaluate the training effects. According to the classification proposed by Hopkins et al. (2009), an ES of less than 0.2 was considered trivial, 0.2–0.6 was small, 0.6–1.2 was moderate, 1.2–2.0 was large, 2.0–4.0 was very large, and greater than 4.0 was nearly perfect. Within-group ES were calculated to quantify the magnitude of change from pre-to post-intervention. ESs were computed as the mean difference divided by the pooled standard deviation and interpreted according to established thresholds. Hedges’ *g* was used to calculate ES for all outcome measures, as this statistic corrects for small-sample size. In addition, between-group differences (i.e., WV-PJT and EB-PJT) were quantified using the standardized mean difference (SMD). SMDs were calculated as the difference in post-intervention mean values between groups divided by the pooled standard deviation (Arazi et al., 2018). Percentage changes from pre-to post-intervention in the variables were calculated using the formula ([post - pre]/pre x 100) and analyzed by one-way ANOVA among the training groups. To assess the reliability of performance variables, intra-class correlation (ICC) was calculated. The significance level was set at 0.05 (Ning and Sheykhlouvand, 2025).

## 3 Results

All players fully complied with the research protocol, achieving a 100% success rate. Moreover, there were no recorded instances of injuries related to the training and assessment methods. Additionally, no statistically significant differences (p > 0.05) were noted between the groups during the initial assessment in anthropometric variables as well as physical and physiological performance. No significant changes were identified in the CG across any physical and physiological variables in the post-assessment. A significant group-by-time interaction (p = 0.001) was found in all physical and physiological measures, indicating that the training groups (WV-PJT, EB-PJT, and UL-PJT) showed more gains than the CG after the training period. These differences highlight that an 8-week volleyball training program alone is not sufficient to elicit improvements in these variables, and further demonstrate that the specific training methods employed in the WV-PJT, EB-PJT, and UL-PJT groups yielded greater improvements compared to the CG.

**FIGURE 2:**
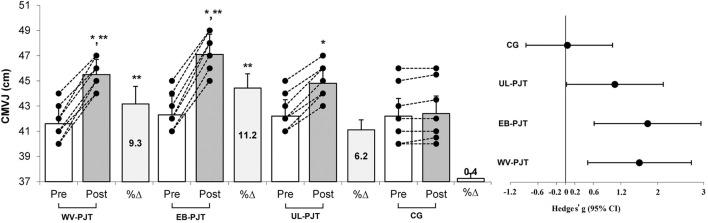
Changes in CMVJ following the 8-week PJT. *Significant differences compared with pre and CG, **significant differences compared with UL-PJT.

**FIGURE 3:**
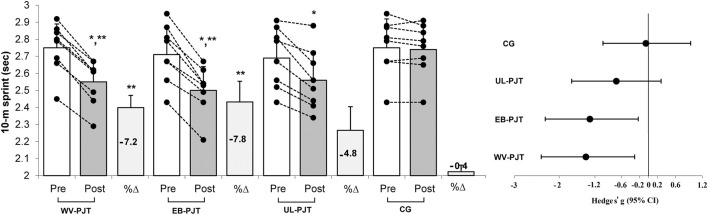
Changes in 10-m sprint following the 8-week PJT. *Significant differences compared with pre and CG, **significant differences compared with UL-PJT.

**FIGURE 4:**
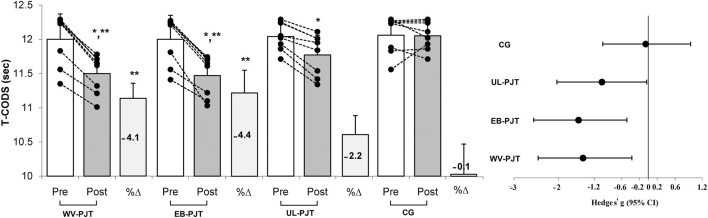
Changes in T-CODS following the 8-week PJT. *Significant differences compared with pre and CG, **significant differences compared with UL-PJT.

**FIGURE 5:**
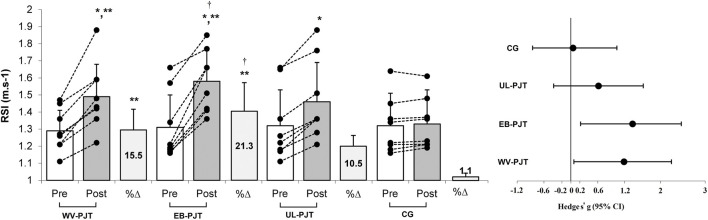
Changes in RSI sprint following the 8-week PJT. *Significant differences compared with pre and CG, **significant differences compared with UL-PJT, †significant differences compared with WV-PJT.

**FIGURE 6:**
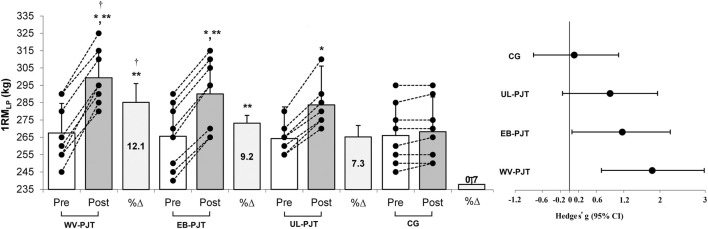
Changes in 1RM_LP_ following the 8-week PJT. *Significant differences compared with pre and CG, **significant differences compared with UL-PJT, †significant differences compared with EB-PJT.

**FIGURE 7:**
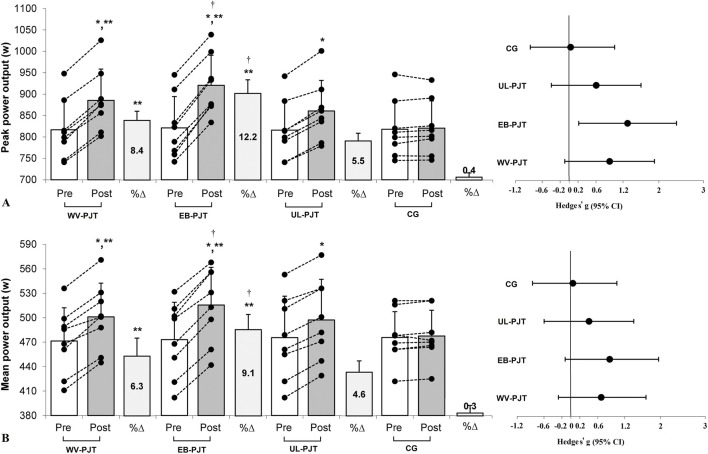
Changes in **(A)** peak power output and **(B)** mean power output following the 8-week plyometric jump training (PJT). *Significant differences compared with pre and control group (CG); **significant differences compared with unloaded PJT (UL-PJT); ^†^significant differences compared with weighted-vest PJT (WV-PJT).

There was significant group × time interaction which indicates greater adaptive changes for the WV-PJT and EB-PJT groups than for the UL-PJT group in the CMVJ (ES = 1.60 and 1.78 vs. 1.07, p = 0.007) (Figure 2), 10-m sprint (ES = −1.40 and −1.31 vs. −0.72, p = 0.009) (Figure 3), T-CODS (ES = −1.44 and −1.54 vs. −1.02, p = 0.001) (Figure 4), RSI (ES = 1.21 and 1.38 vs. 0.62, p = 0.021) (Figure 5), 1RM_LP_ (ES = 1.83 and 1.17 vs. 0.90, p = 0.001) (Figure 6), peak (ES = 1.19 and 1.38 vs. 0.62,p = 0.001) (Figure 7A), and mean (ES = 0.68 and 0.87 vs. 0.41, p = 0.002) (Figure 7B) power output, respectively, following the training period.

There was no significant (p < 0.05) group by time interaction between the WV-PJT and EB-PJT in the CMVJ (p = 0.547), 10-m sprint (p = 0.855) and T-CODS (p = 0.341); however, in the 1RM_LP_ the WV-PJT indicated greater adaptive responses (p = 0.011, 12.1% ± 2.6% vs. 9.2% ± 1.1%, standard mean difference [SMD] = 0.51, 95% CI = −0.51–1.48) than the EB-PJT after an 8-week training intervention period (Table 3). Likewise, the EB-PJT group demonstrated greater adaptations in the RSI (p = 0.041, 21.3% ± 8.8% vs. 15.5% ± 6.4%, SMD = 0.46, 95% CI = −0.53–1.45), peak (p = 0.006, 12.2% ± 1.9% vs. 8.4% ± 1.3%, SMD = 0.49, 95% CI = −0.53–1.46) and mean (p = 0.017, 9.1% ± 1.6% vs. 6.3% ± 1.9%, SMD = 0.33, 95% CI = −0.67–1.30) power output than the WV-PJT after training intervention.

**TABLE 3:** Standard mean difference (SMD with 95% CI) among the PJT groups.

Variables	Groups	SMD	Description
CMVJ (cm)			
	WV-PJT vs. UL-PJT	0.31 (−0.69–1.28)	Small
	EB-PJT vs. UL-PJT	0.89 (−0.14–1.91)	Moderate
	EB-PJT vs. WV-PJT	0.66 (−0.38–1.63)	Moderate
10-m sprint (sec)			
	WV-PJT vs. UL-PJT	−0.07 (−1.04 to 0.92)	Trivial
	EB-PJT vs. UL-PJT	−0.39 (−1.35 to 0.62)	Small
	EB-PJT vs. WV-PJT	−0.37 (−1.35 to 0.64)	Small
T-CODS (sec)			
	WV-PJT vs. UL-PJT	−0.95 (−1.92 to 0.13)	Moderate
	EB-PJT vs. UL-PJT	−1.02 (−2.00 to 0.07)	Moderate
	EB-PJT vs. WV-PJT	−0.10 (−1.08 to 0.88)	Trivial
RSI (m.s-1)			
	WV-PJT vs. UL-PJT	0.14 (−0.85–1.12)	Trivial
	EB-PJT vs. UL-PJT	0.58 (−0.45–1.55)	Small
	EB-PJT vs. WV-PJT	0.46 (−0.53–1.45)	Small
1RMLP (kg)			
	WV-PJT vs. UL-PJT	0.81 (−0.25–1.78)	Moderate
	EB-PJT vs. UL-PJT	0.29 (−0.71–1.26)	Small
	WV-PJT vs. EB-PJT	0.51 (−0.51–1.48)	Small
Peak power output (w)			
	WV-PJT vs. UL-PJT	0.34 (−0.66–1.31)	Small
	EB-PJT vs. UL-PJT	0.85 (−0.22–1.82)	Moderate
	EB-PJT vs. WV-PJT	0.49 (−0.53–1.46)	Small
Mean power output (w)			
	WV-PJT vs. UL-PJT	0.08 (−0.90–1.06)	Trivial
	EB-PJT vs. UL-PJT	0.38 (−0.63–1.35)	Small
	EB-PJT vs. WV-PJT	0.33 (−0.67–1.30)	Small

## 4 Discussion

This study aimed to investigate two main aspects: (a) the effects of PJT on the physical and physiological performance of volleyball players, and (b) the comparison between two different loading strategies—WV (constant load) and EB (variable load)—in maximizing training adaptations in male volleyball players. The main findings indicated that PJT is an effective training method for enhancing physical and physiological performance compared with a CG that only performed volleyball-specific training. Moreover, both loaded PJT conditions (WV-PJT and EB-PJT) elicited greater adaptations than UL-PJT across most measured variables. Specifically, WV-PJT was more effective for improving maximal strength, whereas EB-PJT induced superior improvements in RSI, peak and mean power outputs.

Previous experimental and review studies have consistently demonstrated that bio-motor abilities such as CMVJ, 10 m sprint, and change-of-direction speed can be significantly enhanced following PJT interventions lasting 6–10 weeks in trained and young volleyball athletes (Silva et al., 2019; Ramirez-Campillo et al., 2020; Hernandez-Martinez et al., 2023). These adaptations are primarily attributed to neuromuscular mechanisms, including increased neural drive to agonist muscles, enhanced motor unit recruitment and firing frequency, improvements in inter- and intramuscular coordination, and favorable modifications in muscle–tendon unit stiffness (Saez de Villarreal et al., 2009; Silva et al., 2019). In addition, structural adaptations such as increases in muscle cross-sectional area and alterations in muscle architecture (e.g., pennation angle and fascicle length) may further contribute to improvements in force and power production (Chu, 1998; Wang et al., 2023). The observed improvements in CMVJ performance may also partially explain the concurrent enhancements in sprint and change-of-direction abilities, as vertical force production capacity has been shown to be strongly associated with horizontal acceleration and directional changes in volleyball players (Ramirez-Campillo et al., 2021; Arazi et al., 2018).

Importantly, the present findings indicate that incorporating additional external loads during PJT—via either WV or EB—elicits greater adaptations than unloaded PJT alone, which is consistent with previous interventions conducted in trained athletes over six to 8 weeks (Freitas-Junior et al., 2019; Aloui et al., 2020). From a physiological perspective, loaded PJT likely enhances the capacity of the muscle–tendon unit to store elastic energy during the rapid eccentric loading phase of the SSC and to reutilize this stored energy during the subsequent concentric phase, thereby augmenting concentric force and power output (Aloui et al., 2021). Furthermore, higher internal mechanical loads imposed on the lower-limb musculature may increase recruitment of fast-twitch motor units and elevate the mechanical stimulus required for additional neuromuscular adaptations, ultimately resulting in greater improvements in CMVJ, sprint, and change-of-direction performance (Khlifa et al., 2010).

Given the importance of minimizing ground contact time and optimizing take-off efficiency in volleyball-specific movements (Wagner et al., 2009), RSI represents a sensitive indicator of SSC function and explosive performance. The present study demonstrated that PJT significantly improved RSI, which aligns with previous systematic reviews and experimental studies reporting meaningful RSI gains following 6–10 weeks of PJT in trained populations (Ramirez-Campillo et al., 2018; Rebelo et al., 2022). These improvements are largely explained by neuromuscular adaptations that enhance the rapid transition from eccentric to concentric muscle actions, including increased pre-activation of muscles, enhanced stretch reflex sensitivity, improved musculotendinous stiffness, and greater rate of force development (Chu, 1998; Ramirez-Campillo et al., 2021; Silva et al., 2019). From a motor learning perspective, the high specificity of plyometric drills—performed with maximal intent and short contact times—likely facilitates efficient transfer to RSI performance by reinforcing task-specific neuromuscular patterns (Stone et al., 2002; Davies et al., 2015).

When comparing loading strategies, both WV-PJT and EB-PJT induced greater RSI improvements than UL-PJT, supporting the notion that additional external loading further enhances SSC efficiency and neuromuscular demand (Kang, 2018; Aloui et al., 2021). Interestingly, EB-PJT resulted in superior RSI adaptations compared with WV-PJT after the 8-week intervention. This finding may be attributed to the variable resistance profile of elastic bands, which progressively increases load throughout the range of motion, thereby intensifying eccentric–concentric coupling and neuromuscular activation (Stankovic et al., 2025; Aloui et al., 2020). Studies using variable resistance have reported greater motor unit recruitment, enhanced rate coding, and increased myoelectric activity compared with constant loads, suggesting a heightened neuromuscular stimulus (Ali et al., 2024; Iranpour et al., 2025; Gaamouri et al., 2023). Nevertheless, as neuromuscular activity was not directly assessed, these interpretations remain speculative and warrant confirmation in future studies incorporating electromyographic analyses.

Consistent with previous research, PJT also induced significant improvements in maximal strength performance (1RM), particularly when additional external loads were incorporated (Silva et al., 2019; Saez de Villarreal et al., 2010). Both WV-PJT and EB-PJT elicited greater strength gains than UL-PJT, likely due to enhanced neural adaptations such as increased motor unit firing frequency, improved synchronization, reduced antagonist co-activation, and heightened neural drive (Saez de Villarreal et al., 2009; Saez de Villarreal et al.,2010). Moreover, musculoskeletal adaptations—including muscle hypertrophy and a shift toward type II muscle fiber characteristics—may further contribute to strength development following loaded PJT (Davies et al., 2015; Arntz et al., 2022).

Notably, WV-PJT resulted in superior improvements in 1RM compared with EB-PJT. This finding may be explained by the overload principle, whereby the constant external load provided by WV enables athletes to generate higher vertical ground reaction forces over a longer portion of the concentric phase during jump tasks (Khlifa et al., 2010). This sustained force application increases net impulse, which likely promotes force-oriented mechanical adaptations and, consequently, greater improvements in maximal strength compared with variable loading conditions (Freitas Junior et al., 2019).

A novel contribution of the present study is the examination of anaerobic power responses to loaded PJT using WV and EB. The findings demonstrated moderate to large improvements in peak and mean power output following PJT, particularly under loaded conditions, with EB PJT eliciting greater gains than WV PJT. Previous research indicates that PJT enhances anaerobic power primarily through neuromuscular mechanisms, including greater recruitment of high-threshold motor units, increased firing rates, and improved efficiency of the phosphagen system (Malisoux et al., 2006; Markovic and Mikulic, 2010; Song and Sheykhlouvand, 2024). Collectively, these adaptations contribute to improvements in peak and mean power output. The superior anaerobic power adaptations observed following EB PJT may be attributed to the greater metabolic and neuromuscular demands imposed by variable resistance throughout the range of motion (Aloui et al., 2020). This loading profile may provide a more effective stimulus to peripheral metabolic pathways, potentially enhancing phosphagen system efficiency, metabolite clearance, and the maintenance of ATP and PCr stores during high-intensity efforts (Stankovic et al., 2025; de Poli et al., 2021; Gaamouri et al., 2023; Gharaat et al., 2020; Li and Sheykhlouvand, 2025; Forbes and Sheykhlouvand, 2016). However, as metabolic and neuromuscular variables were not directly assessed in the present study, these proposed mechanisms remain speculative and should be confirmed in future investigations.

Despite the practical relevance of these findings, several limitations should be acknowledged. The relatively small sample size (n = 8 per group) may limit generalizability, although an *a priori* power analysis indicated sufficient statistical power (Freitas-Junior et al., 2019). Additionally, the exclusive inclusion of male volleyball players restricts extrapolation to female athletes or other sports populations. The absence of direct assessments of neuromuscular (e.g., EMG) and metabolic adaptations further limits mechanistic interpretation. Importantly, although improvements were observed in physical and physiological performance, the transfer of these adaptations to actual competitive volleyball performance remains uncertain due to the lack of systematic match or time-motion analysis. Future studies incorporating match-based performance indicators and biomechanical monitoring are required to clarify the ecological validity of PJT-induced adaptations.

## 5 Conclusion

The findings of this study indicate that integrating an 8-week PJT program, performed three times per week in conjunction with regular technical–tactical volleyball training, effectively enhances physical performance and anaerobic power output in male volleyball players. Notably, PJT performed with additional external loads elicited superior adaptations compared with unloaded PJT. Specifically, the WV-PJT was more effective for improving maximal strength, whereas EB-PJT produced greater enhancements in RSI and anaerobic power output. Practically, both WV and EB loading strategies can be periodized within a season according to specific performance objectives, athlete readiness, and training phase. Overall, the use of loaded PJT represents an effective, time-efficient, and sport-specific approach for improving bio-motor abilities and anaerobic performance in male volleyball players. From a practical perspective, coaches and practitioners are encouraged to incorporate loaded PJT into volleyball training programs to optimize performance adaptations. During phases in which maximal strength development is prioritized (e.g., early pre-season or strength-oriented mesocycles), WV-PJT appears to be the most appropriate strategy. Conversely, when the primary goal is to enhance explosive qualities requiring rapid force production, such as RSI and anaerobic power (e.g., late pre-season or competitive phases), EB-PJT may be preferable.

## Data Availability

The raw data supporting the conclusions of this article will be made available by the authors, without undue reservation.
